# Wearable Crop Sensor Based on Nano-Graphene Oxide for Noninvasive Real-Time Monitoring of Plant Water

**DOI:** 10.3390/membranes12040358

**Published:** 2022-03-24

**Authors:** Denghua Li, Ganqiong Li, Jianzheng Li, Shiwei Xu

**Affiliations:** 1 Agricultural Information Institute of Chinese Academy of Agricultural Sciences, Beijing 100081, China; lidenghua@caas.cn (D.L.); lijianzheng@caas.cn (J.L.); 2 Key Laboratory of Agricultural Monitoring and Early Warning Technology, Ministry of Agriculture and Rural Affairs, Beijing 100081, China; 3 Research Center of Agricultural Monitoring and Early Warning Engineering Technology, Beijing 100081, China

**Keywords:** graphene oxide, sensor, crop water, noninvasive, monitoring

## Abstract

Real-time noninvasive monitoring of crop water information is an important basis for water-saving irrigation and precise management. Nano-electronic technology has the potential to enable smart plant sensors to communicate with electronic devices and promote the automatic and accurate distribution of water, fertilizer, and medicine to improve crop productivity. In this work, we present a new flexible graphene oxide (GO)-based noninvasive crop water sensor with high sensitivity, fast responsibility and good bio-interface compatibility. The humidity monitoring sensitivity of the sensor reached 7945 Ω/% RH, and the response time was 20.3 s. We first present the correlation monitoring of crop physiological characteristics by using flexible wearable sensors and photosynthesis systems, and have studied the response and synergistic effect of net photosynthetic rate and transpiration rate of maize plants under different light environments. Results show that in situ real-time sensing of plant transpiration was realized, and the internal water transportation within plants could be monitored dynamically. The synergistic effect of net photosynthetic rate and transpiration of maize plants can be jointly tested. This study provides a new technical method to carry out quantitative monitoring of crop water in the entire life cycle and build smart irrigation systems. Moreover, it holds great potential in studying individual plant biology and could provide basic support to carry out precise monitoring of crop physiological information.

## 1. Introduction

Water is the lifeblood of agriculture and plays an important role in plant photosynthesis, transpiration, and temperature regulation; the growth rate of plants is significantly affected by water status [1]. Dynamic and accurate monitoring of crop water and intelligent on-demand supply is very important to improve crop productivity. According to the existing water use efficiency, the water for agricultural irrigation will increase by approximately 36% by 2030, and the shortage of water resources has become the main restrictive factor for global food security [2,3]. In the traditional agricultural production mode, irrigation mostly depends on the experience and judgment of farmers. Flood irrigation leads to a large waste of water in agricultural production. Thus, smart irrigation technology should be developed urgently for the efficient utilization of water resources. Intelligent sensors and information monitoring are the bases and the “five senses” of smart agriculture. Therefore, developing accurate and efficient crop water monitoring sensors and smart irrigation equipment is greatly important.

Conventional technologies, such as remote sensing spectral imaging [4], infrared fluorescence [5], Raman spectroscopy [6] have been developed for monitoring plant physiological information. However, these techniques have low cost-benefit and are vulnerable to low-noise signals and interfering weather conditions, and the accuracy and timeliness are insufficient [7]. Pressure chambers are a widely accepted plant-based method, but they are unsuitable for automatic monitoring systems because the technology is slow and invasive to plants [8]. Rapid and continuous tracking of a plant’s internal physiological traits remains a challenge for the current silicon-based sensing technologies, due to the mismatch between the rigid sensor and the soft bio-organism, which can either cause damage to the plant or intervene in its normal physiological process. Therefore, although these technologies have played an important role in plant information monitoring, their practical application still has many deficiencies, including discontinuous monitoring, low sensitivity/specificity, high cost, and time/labor consuming, far below the requirements for smart agriculture. Therefore, developing new methods that can monitor, track, and predict plant water and health status in real-time with high reliability and improved signal-to-noise ratio is urgent. This method will help to continuously monitor crop water at the plant and leaf levels to timely optimize and adjust plant growth conditions and improve crop productivity.

New opportunities to address these problems emerged recently owing to the tremendous advances in flexible electronic technology [9,10]. Many efforts had been exerted on the research of developing plant bioelectronics sensors based on nanofilms, which convert plant life related chemical signals into electrical signals effectively [11,12]. The integration of nano-electronic technology and plant science provides a new approach for the digital and accurate monitoring of plant water and other life information. Key performance indicators, such as linearity and sensitivity of the sensor, are mainly affected by the sensing materials. Effective materials should be explored for the plant physiological water sensing. Graphene oxide (GO) has shown great potential for detecting humidity due to its unique two-dimensional nanostructure and huge specific surface areas. GO based sensors have attracted the attention and favor of researchers and were widely used in gas detection [13], toxicity detection [14], biosensor [15], and mechanical sensing [16,17]. GO has been widely considered a compromise between the interesting properties of graphene and the synthesis price and complexity. Large numbers of oxygen-containing functional groups in the GO nanolayers interact with the water molecules of environmental objects easily, making it an ideal candidate for humidity sensing [18,19]. Despite a few successful examples of monitoring external characteristics, such as the temperature and humidity on the leaf surface, and growth of plant organs [20,21,22], there is still a great challenge to develop noninvasive wearable crop physiological sensors with high sensitivity, fast response–recovery rate, and ultralow limit of detection. Moreover, studies on the correlation monitoring between wearable sensors and key physiological information (e.g., photosynthesis information) are limited and have not been reported yet. Therefore, monitoring the communication between plant physiological behaviors and their surrounding environment is greatly valuable, as well as analyzing the correlation between plant water information and photosynthesis performance.

In this study, we present a wearable resistive-type crop water sensor based on nanomaterial that can harmlessly cohabitate with the plant and continuously track the water transportation in the plant, which is one of the most critical physiological characteristics for a plant, indicating the growth status, water consumption, and photosynthetic intensity [23]. We first present the correlation monitoring of crop physiological characteristics by using flexible wearable sensors and photosynthesis instruments and have studied the response and synergistic effect of net photosynthetic rate and transpiration rate of maize plants under different light environments. By taking advantage of the GO nanomaterials and nanofabrication, our sensor has high sensitivity, fast response rate and biocompatibility. We believe that our work holds great potential in studying individual plant biology, and could provide basic support to carry out precise monitoring of crop physiological information and build smart planting systems.

## 2. Materials and Methods

### 2.1. Materials

The reagents used in the experiment are absolute ethanol, concentrated sulfuric acid (98%), KMnO_4_, H_2_O_2_ (5%) HCl, deionized water, acetone, and isopropanol. The maize plants (Huawei 6, Beijing Baofeng Seed Co., Ltd., Beijing, China) were grown in an artificial climate chamber of the Agricultural Information Institute of Chinese Academy of Agricultural Sciences.

### 2.2. Preparation of GO

GO was prepared by oxidizing graphite powder based on a modified Hummers method reported elsewhere [24,25]. Typically, 1 g of natural graphite was mixed with 0.5 g of sodium nitrate and added to 23 mL of cold concentrated H_2_SO_4_ in an ice water bath. KMnO_4_ (3 g) was added gradually with vigorous stirring for 2 h, and the temperature of the mixture remained below 20 °C. The mixture was transferred into a constant temperature water bath at 35 °C and continuously stirred for 0.5 h. Distilled water (80 mL) was slowly added, and the mixture was allowed to remain at 90–98 °C for 15 min. Then, 5% H_2_O_2_ was added to the solution until the solution turned bright yellow. The mixture was filtered using a Buchner funnel, and the solid material was washed with 5% HCl and excess deionized water until the filtrate obtained a pH value of 7. Finally, the filter cake (GO) was dried in a vacuum oven at 100 °C for 12 h and stored until use. Then 50 mg GO powder was added into deionized water (10 mL); the reagent bottle was mixed by shaking and then dispersed by an ultrasonic instrument for 1 h. The ultrasonic frequency was 37 kHz, and the ultrasonic power was 100 W to prepare a stably dispersed GO solution (5 mg/mL).

### 2.3. Preparation of Polyimide (PI) Flexible Substrate Sensor

**Figure 1:**
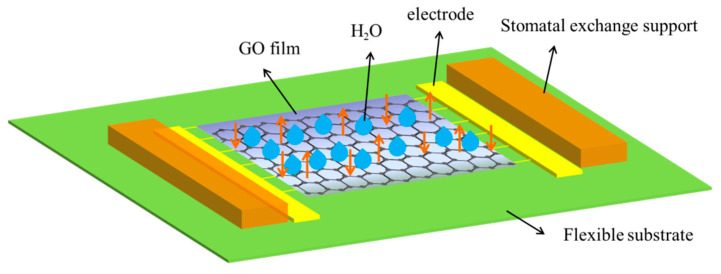
Schematic of the structure of the flexible devices.

The preparation of the interdigital electrode structure was completed by MEMS technology. Chromium film with a thickness of 20 nm and gold film with a thickness of 100 nm was deposited on a PI flexible substrate. First, the PI substrate was ultrasonically cleaned in acetone solution and ethanol solution to remove metal ion pollution and particle pollution, and then the water on the surface of the glass sheet was blown off with dry and clean N_2_ and fully dried in the drying oven. Second, a layer of uniformly covered chromium and gold film was evaporated on the PI substrate; 20 nm thick chromium film and 100 nm thick gold film were grown on the insulating substrate by magnetron sputtering. Third, a uniform layer of photoresist was spin coated on the substrate surface by a glue homogenizer, and then the UV exposure process was carried out on the substrate surface by using the photoresist and the photoresist mask plate. Then, the mask pattern was developed in the developing solution. After photolithography, the electrode pattern of the photoresist was formed on the surface of the chromium gold metal film. Finally, the device was wet etched, and the interdigital electrode pattern was obtained. The line width and line distance of the interdigital electrode were 50 μm. GO ink drops were dipped on the interdigital electrode substrate by dip coating method to form a thin film, which was annealed at 110 °C for 1 h. To facilitate the installation of the sensor onto the leaf surface, two stomatal vapor exchange supports were adhered to the two sides of the graphene pattern, thereby creating an approximately 500 μm thick air gap between the sensitive layer and the leaf. The air gap allows air exchange between inside and outside of the gap space to avoid water vapor accumulation. The main structure diagram of the device is shown in Figure 1.

### 2.4. Characterizations and Testing

The surface microstructure and morphology of the prepared materials were investigated using a field emission scanning electron microscope (SU8020, Hitachi, Tokyo, Japan). The humidity sensing properties were investigated by exposing the fabricated sensor in various relative humidity (RH) values, which were achieved by several saturated aqueous solutions. Saturated solutions of LiCl, MgCl_2_, Mg(NO_3_)_2_, NaCl, KCl, and KNO_3_ in a closed vessel were used to obtain 11%, 33%, 54%, 75%, 85%, and 95% RH levels, respectively. The experiments were performed at room temperature.

The electrical properties of the proposed sensor prototype under various RH levels were measured using a precision electrochemical workstation (ECW-SA5120S, SINO AGGTECH, China). Sensitivity (S), which was used to characterize the sensor performance, was defined as S = ΔZ/ΔRH (unit: Ω/%RH), where ΔZ is the sensor response change in impedance, and ΔRH is the RH change. The hysteresis error was defined as a deviation due to hysteresis between upscale-going and downscale-going indications in terms of the full measured quantity. The sorption/desorption hysteresis of humidity sensors was studied by sequentially exposing sensors to 11%, 33%, 54%, 75%, 85%, and 95% RH for water sorption and then to 85%, 75%, 54%, 33%, and 11%RH for desorption of water. The exposure time at each relative humidity was 10 min. The time required by a sensor to achieve 90% of the total resistance change was defined as the response or recovery time.

The impedance of the sensor mounted on plants was measured using an LCR meter (Wayne Kerr, 4100, Wayne Kerr Electronics, London, UK), which was connected to a PC through a LAN interface for data acquisition. The light response curve of photosynthesis was measured by a portable photosynthesis measurement system (LI-6400, LI-COR Inc., Lincoln, NE, USA).

## 3. Results and Discussion

### 3.1. Characterization of Sensing Films

**Figure 2:**
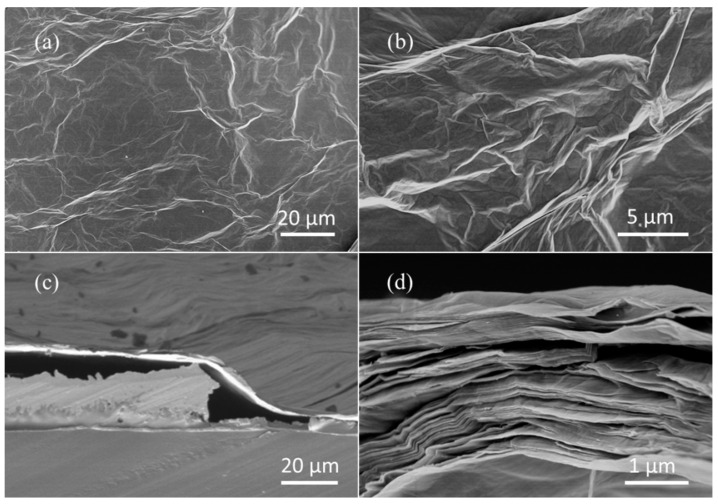
SEM micrographs of the sensitive films. (**a**,**b**) top-view surface micrographs; (**c**,**d**) cross section micrographs.

Morphology and defect characteristics of the film were investigated. This is helpful to analyze the sensitive property, mechanism and characteristics of the film. This is also helpful to guide the optimization of the preparation of sensitive films and improve the sensing performance. The microscopic structure of the as-synthesized GO films was explored using the SEM image in Figure 2. Figure 2a,b shows the surface of the sensitive films, indicating that GO has a typical layered and wrinkled silk-like structure. An ultrathin sheet structure with few thin ripples within the plane can be observed, reflecting a uniform 2D structure of the carbon sheets [26]. The highly wrinkled structure of GO films is expected to be effective for high sensitivity and increasing the surface area. Elemental analysis of the material film by Energy Dispersive Spectrometer (EDS) shows that the content percentages of elements C and O were 60.1% and 36.4%, respectively (Figure S1). The high content of oxygen indicates that the functional groups are rich. Thus, the GO sheets become more hydrophilic and humidity sensitive. Figure 2c,d shows a cross section of the sensitive films, presenting the layer-by-layer sheets attached to the interdigital electrode and flexible substrate. The thickness of the sensitive films is approximately 2 μm from their cross section images. A thin film sensor is proposed by taking advantage of the excellent processability of GO aqueous dispersion. According to previous studies, a humidity sensor based on a film sorbs and de-sorbs water through different processes; the former can be ascribed to the “surface adsorption” which is determined by the properties and surface morphology of thin films. After the surface process, other slower sorption/desorption mechanisms, such as diffusion or relaxation, occur. In most cases, the response time of the system is determined, and it is strongly dependent on the thickness of the sensitive film [27]. Therefore, a sensor with a large specific surface area and a thin film is expected to exhibit a faster response. The quality and defect condition of GO films were investigated and compared with rGO films by Raman spectroscopy experiments (Figure S3 of the Supporting Information). This technique is a powerful tool for investigating ordered and disordered nanocarbons. The Raman spectrum of GO and rGO films were characterized by the presence of bands at 1350 cm^−^^1^ (D band) and 1580 cm^−^^1^ (G band). *I_D_*/*I_G_* ratios (where *I_D_* and *I_G_* are the D-band and G-band Raman intensities) are widely used to evaluate the quality of carbon materials [28]. As shown in Figure S2, the *I_D_*/*I_G_* ratio of GO was 0.97, and the *I_D_*/*I_G_* ratio of rGO was 0.60. This implies that the defect ratio of GO is much higher than that of rGO. Although there are many defects in GO films and the conductivity is not high in dry air, the defects in the films played an important role in the adsorption of water molecules. The molecular interactions between the increased water and charged groups in GO would make the GO sensor have high proton-conductive sensitivity to water molecules [29].

### 3.2. Sensor Properties and Impedance Analysis

**Figure 3:**
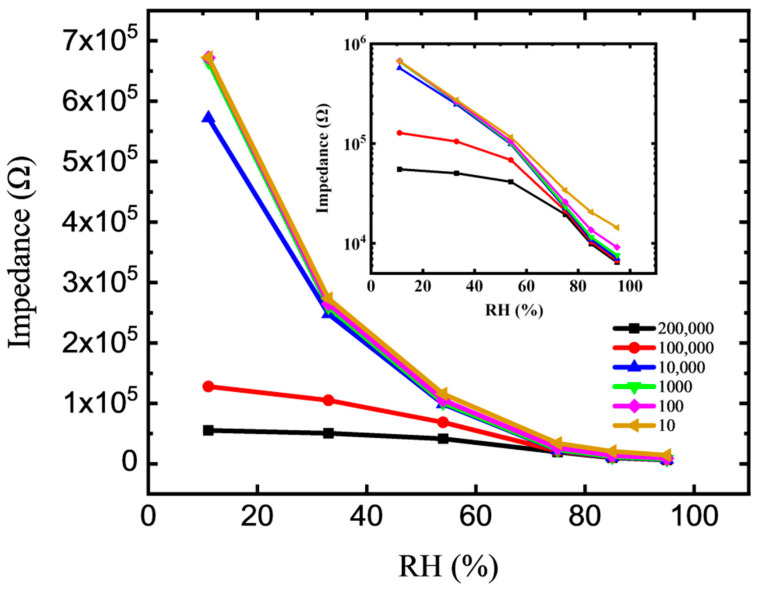
Impedance versus RH properties of the GO-based sensor. The insert is the impedance–RH curves displayed in logarithmic coordinates.

To study the humidity sensing performance and sensitivity of the device, the impedance of the device in a saturated salt solution with different relative humidity and test frequencies was measured. Figure 3 shows the impedance–RH properties of the sensor with a voltage of 0.2 V, and different measured frequencies of 10 Hz to 200 kHz. As the RH level increases, the impedance of the sensor decreases monotonically. The mechanism supporting the sensitivity of GO to humidity may be the result of electrostatic interactions between water and GO [30]. This finding can be interpreted as follows: the absorbed water molecules are beneficial to strengthen the polarization effect and increase the conductivity. This can be attributed to the fact that GO film not only has a high surface area volume ratio but also has a large surface vacancy density and a large number of oxygen-containing functional groups, including carboxyl and hydroxyl groups. The frequency evidently influences the impedance–RH property, especially in the low RH range. Corresponding to the same relative humidity, the impedance value decreases with the increase in frequency. This condition could be due to the water molecules that cannot be polarized at high frequency. The polarization speed of the water molecules cannot cope with the rapid electrical field changes in the high-frequency region, resulting in the reduction of the resistance characteristics of the device [31,32]. As shown in the inset of Figure 3, the best linearity of the impedance–RH curve appears at the frequency of 100 Hz. Thus, the frequency of 100 Hz is selected for the measurement of sensitivity and response time in this work. The humidity sensing characteristics of the devices are different under different frequency conditions. The impedance of the device is greatly affected by the frequency in the low humidity section (<50% RH) and almost coincides with the high humidity section.

**Figure 4:**
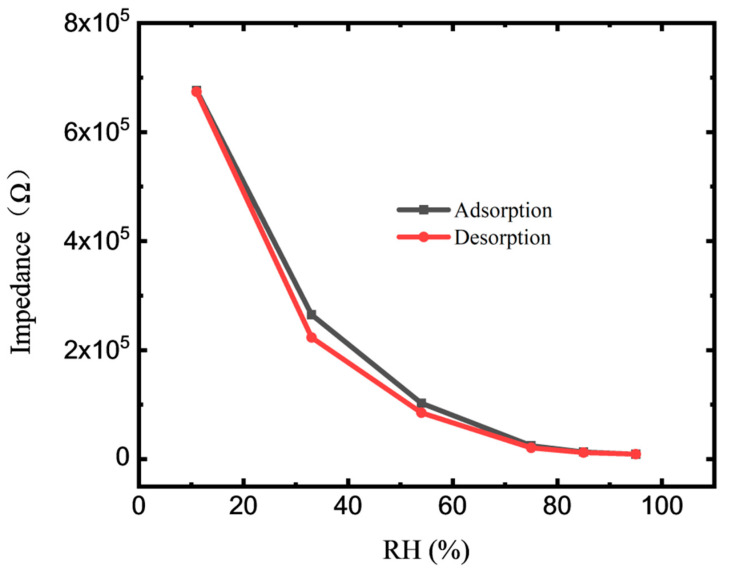
Hysteresis characteristic of the GO-based sensor at 100 Hz.

The hysteresis of humidification and desiccation measured over an RH range of 11–95% RH is shown in Figure 4. The measured voltage and frequency are 0.2 V and 100 Hz, respectively. The black curve in the figure indicates the adsorption process (measured from low to high RH), and the red curve indicates the desorption process (measured in the opposite direction). The resistance changes between 6.7 × 10^5^ and 9.2 × 10^3^ with respect to the RH range of 11–95%, showing the high sensitivity of the sensor. The impedance of the sensor decreases by approximately two orders of magnitude with increasing RH from 11% to 95%. The humidity monitoring sensitivity of the sensor reached 7945 Ω/% RH. The excellent humidity sensing performance of GO film is due to its large number of hydrophilic groups, and vacancies are chemically attached to the defect or the edge sites on basal planes [33]. The response of the element impedance in semi logarithmic coordinates shows good linearity (Figure S3), and the maximum humidity hysteresis of the sensor is approximately 5.3% RH. This result indicated that the sensor shows good reliability and repeatability.

**Figure 5:**
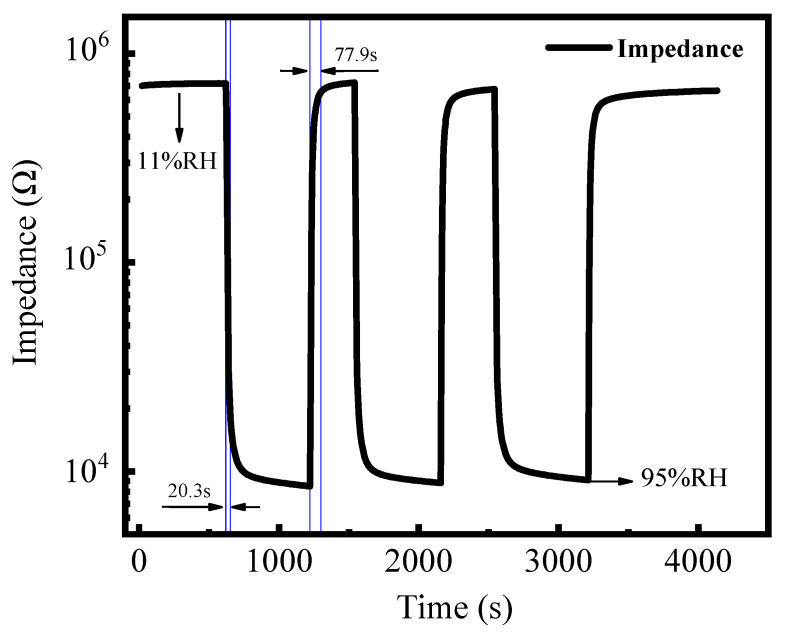
Dynamic response–recovery curves of RH sensitivity of the GO-based sensor at room temperature.

The response recovery characteristic is an important parameter to evaluate the performance of the sensor. The response recovery speed of the device from 11% RH to 95% RH humidity environment was measured. Figure 5 shows the response–recovery characteristics of the sensor measured at 0.2 V and 100 Hz. The response and recovery times represent the time required for the sensor to achieve 90% of the total impedance change when the fabricated sensor was exposed to the target gas [34]. The response time from 11% RH to 95% RH of the device at room temperature is approximately 20.3 s, and the recovery time is slightly longer, approximately 77.9 s. As the response and recovery time is strongly dependent on the thickness of the sensitive film, and the film thickness prepared in this work is micron level (2 μm, Figure 2d) but not nano level, the response speed of the sensor may be affected. As expected, a faster response would be obtained by preparing thinner sensing layers [35]. The response time is shorter than the recovery time meaning it takes a longer time to desorb water than to absorb it into the sensing layer. The reason for the formation of moisture stagnation may be the energy required for the two processes of moisture absorption and dehumidification is different. The water molecules adsorbed on the surface of sensitive materials may require more energy to separate from the element surface. Therefore, the element resistance cannot return to the resistance value during moisture absorption at the same time. These results agree well with previous reports [36,37]. In addition, the reversible cycles of the sensor show that the impedance can be fully restored to the initial impedance value and confirmed that the sensor possessed acceptable reproducibility at room temperature. Fifteen-cycle response-recovery experiments of GO based sensors were conducted to verify the sensing repeatability (Figure S4 of the Supporting Information). The long-term stability of the sensor was measured for 21 days (Figure S5). As shown in Figure S5, both the impedance and capacitance of the sensor show minor variation in the long term, implying acceptable long term stability of this sensor.

### 3.3. Sensing Mechanism of the GO Layered Films

**Figure 6:**
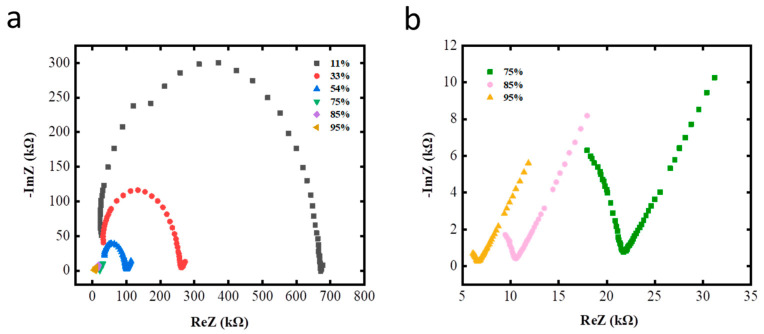
Complex impedance plots of the GO-based sensor at (**a**) RH values from 11–95%, (**b**) RH values from 75–95%. ImZ: imaginary part of complex impedance; ReZ: real part of complex impedance.

Analyzing the complex impedance spectrum of materials could help analyze the sensitive mechanism of materials. To analyze the different sorption mechanisms at various RH values, complex impedance spectra (CIS) were adopted to interpret the conductivity processes that occurred in the sensor. GO sensors were tested in the humidity bottle with different RH values. The real part, imaginary part, and complex angle of its complex impedance at different frequencies were measured, and then the complex impedance curve with the real part of the complex impedance value as the horizontal axis and the imaginary part as the vertical axis was plotted. Figure 6 shows the semi-circular CIS curves at various RH values. As shown in Figure 6a, the complex impedance diagram of the humidity sensor is mainly semi-circular at low RH values of 11% RH and 33% RH. Alongside continuously increasing RH to 75% RH, the semi-circular behavior observed in the low-frequency range is depressed and a straight line appears. When the humidity increases to 85% RH and 95%, the semi-circular structure of the complex impedance diagram almost disappears and changes into a straight line (as shown in Figure 6b). The shape of the complex impedance curve changes with the change in relative humidity, indicating that the conductive mechanism changes accordingly under a different relative humidity. The sensor has different conductivity types, that is, different types of carriers under different humidity conditions. The semicircle is typical of the relaxation mechanism exhibited by resistance–capacitance parallel circuits. Therefore, the semicircle results primarily from the intrinsic impedance of the material [38]. The curvature of the semicircle decreases as the RH increases, thereby reflecting the decrease in the intrinsic impedance and increase in interaction between the sensing film and water [39]. The chemically adsorbed water molecules dissociate at the boundary and defects of GO film to produce protons. The protons and the electrons of the GO material participate in conduction. In addition to resistance and capacitance, diffusion and interface phenomena of ions also play an important role in the physical phenomena of electrical conductivity. Therefore, with the further increase in humidity (>85%), the dominant straight line in the CIS reflects the enhanced ionic conductivity behavior [40], which may stem from the ionic and/or electrolytic conductivity. The water molecules physically adsorbed on the GO surface form a water film and ionize protons. Protons easily combine with water molecules to form hydrated hydrogen ions (H_3_O^+^) when water molecules are abundant. Thus, the main conductive mode of the material at this time would be hydrated hydrogen ion conduction. Full comprehension of the sensing mechanism of the film under various RH revealed here would help to guide the design of materials with better performance, such as higher sensitivity, faster response speed and better stability. The GO based sensor with a high density of active sites and greatly increased interfacial areas between the active sensing regions can significantly improve sensing properties [41]. The sensitive mechanism of ultra-thin graphene film capable of ultrafast induction has been revealed elsewhere [36]. Thus, the fabricated RH sensor with ultrathin GO films and modified functional groups would exhibit the most excellent sensing performance.

### 3.4. In Situ Water Movement Monitoring within Plants

Water transport is a critical process for plants. Crops need water to maintain normal growth, reduce body temperature, and transport nutrients [42]. When plants are subjected to water stress, it affects their hydraulic characteristics and hinders plant water transmission [43]. Therefore, real-time dynamic monitoring of crop water is greatly important to evaluate the adaptability of crops to water stress and adjust the reasonable growth environment of crops. The leaf, as one of the most important organs of plants, is the main place of photosynthesis and transpiration, and the real-time monitoring of its water is particularly important. Water movement within a crop from the roots to the lower and upper leaves was monitored using flexible GO-based RH sensors. The sensing mechanism is based on changes in the electrical impedance of GO in different moisture environments. During transpiration, when the stomata are open, water in the leaves evaporates from the leaf stomata, increasing the local humidity level on the leaf surface. The stomata of most herbs, such as maize, are more distributed on the lower surface of leaves. Consequently, plant transpiration could be monitored in real time by installing sensors on the lower surface of leaves and recording the electrical signals, which do not affect the normal photosynthesis of plants. In addition, by installing multiple RH sensors on different leaves of a plant, the key time point at which significant water transpiration occurs at the leaves could be tracked, quantifying water transport time via the xylem from the roots to each of the measured leaves.

**Figure 7:**
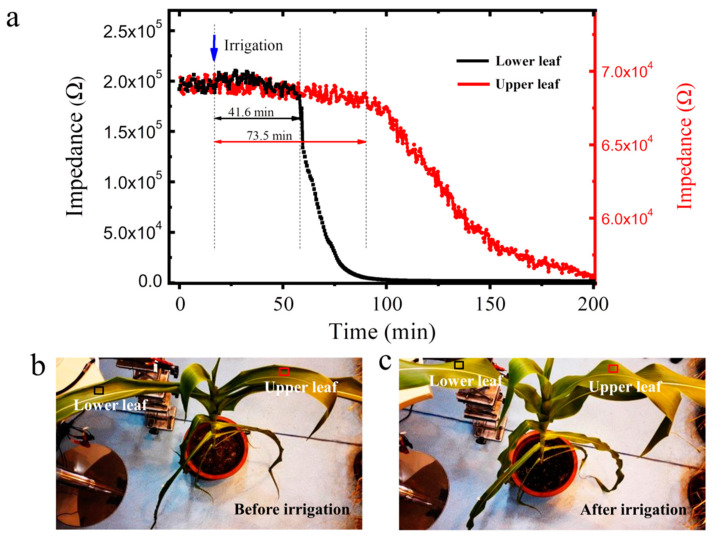
(**a**) Time for water to reach different leaves from the roots after irrigation; (**b**) plant morphology before irrigation; (**c**) plant morphology after irrigation.

Figure 7a shows the real-time monitoring of RH using the sensors installed and attached to the lower surface of the eighth and ninth leaves of maize plants. The black and the red curves are the monitoring data of the sensors installed on the eighth and the ninth leaves, respectively. The testing was initiated 20 min before irrigation. The leaf moisture content of the eighth leaf was 74.2% using the drying and weighing method [44]. The sensor monitoring curve of maize leaves was steady before irrigation, suggesting that the leaf water evapotranspiration was stable. After irrigation, the lower and upper sensors exhibited an impedance decrease and an increase in RH at 41.6 and 73.5 min, respectively. Therefore, less time was required for the water to be transported from the roots to the eighth leaf than from the roots to the ninth leaf, and the time interval is 31.9 min. Thus, the water transport time via the xylem from the roots to each of the measured leaves could be quantified. The maize root system gradually absorbs water in the soil after irrigation. Water is transported from the root to the stem and then flows to the leaves from the bottom to up. The time of water reaching the leaves increases with the distance from the leaves to the roots. When the water reaches the leaves, the cell water flowing to the sensor monitoring area gradually increases, and the transpiration and the concentration of evaporated water vapor gradually increase. Therefore, the impedance of the device gradually decreases. The sensor impedance decreased and gradually stabilized, suggesting that the water transport gradually reached a stable state after reaching the leaf tissue, and the leaf water content was 85.7%. That is, the leaf water content increased by 11.5% after irrigation compared with that before irrigation. Figure 7b,c shows that the leaf tissue of water deficient maize plants showed evident morphological changes before and after irrigation. Water deficit directly affects the physiological and biochemical processes and morphological structure of crops, as well as the crop growth, yield, and quality. The proposed sensor can monitor the transpiration water of crops 24 h a day. By building the Internet of things system, a new technical method is provided for carrying out the quantitative monitoring of crop water in the entire life cycle and building a new intelligent irrigation system in the future.

### 3.5. Correlation Monitoring of Light Regulated Transpiration and Photosynthesis

**Figure 8:**
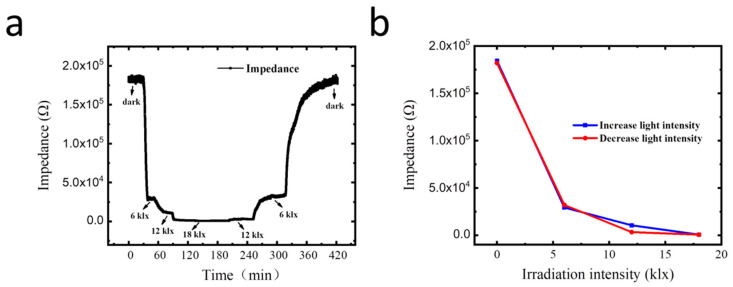
(**a**) Impedance variation of the GO-based sensor attached to a leaf surface as response to light intensity over time; (**b**) measurements of the sensor attached to the lower surface of a leaf under different light irradiation intensities.

In addition to water conditions, light significantly affects the transpiration of plants. The performance of the GO sensor on monitoring light-regulated transpiration was also tested. Figure 8 shows the impedance variation of the sensor attached to the lower surface of a maize leaf as a response to irradiation intensity over time. The maize plant to be tested was placed in the artificial climate chamber and was irrigated normally, and the temperature of the artificial climate chamber was set at 25 °C. As shown in Figure 8a, the impedance decreases rapidly with the increase in irradiation intensity. The impedance was 183.2 kΩ when the light was off and 0.75 kΩ when the lighting was 18 klx, showing 244.3 times changes in the impedance of the sensor. Moreover, when the lighting was gradually turned down until finally turned off, the impedance of the sensor gradually increased accordingly to a stable level of 181.4 kΩ, which is close to the initial impedance value. Figure 8b shows that the impedance decreases dramatically when the irradiation intensity increases from 0 klx to 18 klx, indicating the high sensitivity of the sensor for irradiation-regulated transpiration sensing. According to previous reports, photosynthesis and transpiration of plants share a common path, that is, through the guard cells to adjust the stomatal opening, carbon dioxide diffuses into the leaves and water diffuses out, although they are two relatively independent processes [45,46]. The degree of stomatal opening affects photosynthesis and transpiration [47]. These results suggest that the flexible GO-based RH sensor has potential applications in the real-time monitoring of plant photosynthetic physiological information.

**Figure 9:**
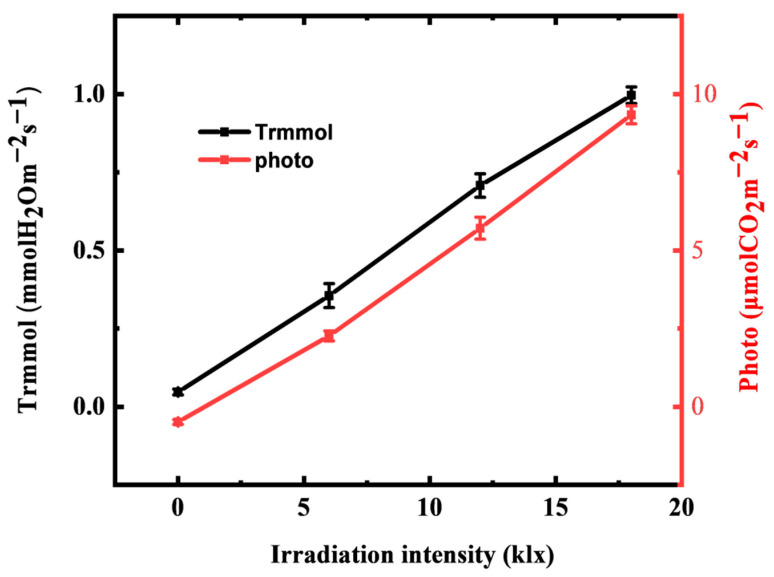
Transpiration rate (Trmmol) and net photosynthetic rate (Photo) of a crop leaf as response to light irradiation intensity.

We used the sensor and portable photosynthesis measurement system to jointly test the response and synergistic effect of net photosynthetic rate (Photo) and transpiration rate (Trmmol) of maize plants under different light environments to investigate the performance of the leaf sensor in the real-time monitoring physiological water information of maize plants. Figure 9 shows the transpiration rate and net photosynthetic rate of a growing crop leaf as a response to the light intensity at 25 °C. When the light intensity increased from 0 klx to 18 klx, the transpiration rate increased from 0.05 mmol m^−2^ s^−1^ to 0.99 mmol m^−2^ s^−1^, and the transpiration rate increased from −0.49 μmol m^−2^ s^−1^ to 9.34 μmol m^−2^ s^−1^. The net photosynthetic rate and transpiration rate of maize increased with the increase in light intensity and showed a linear increasing relationship. However, no light saturation point is found within the light irradiation intensity of 18 klx.

**Figure 10:**
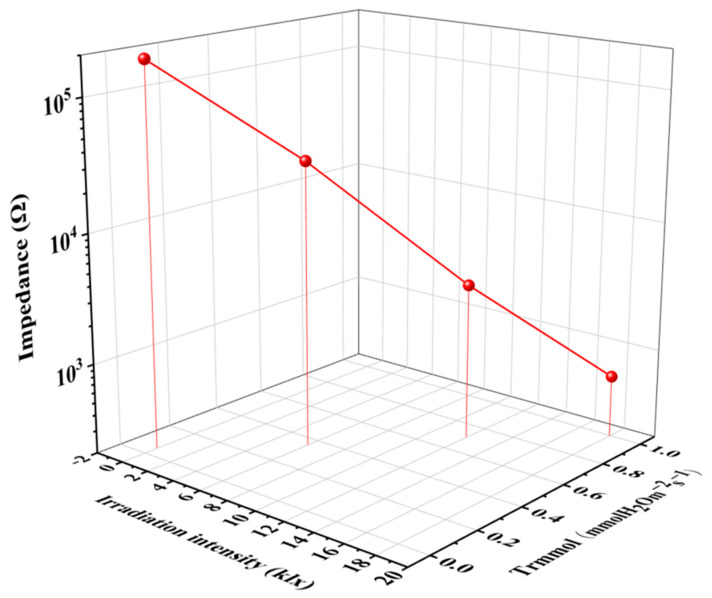
Impedance and transpiration rate (Trmmol) response of GO-based sensor under different irradiation intensities.

Figure 10 displays response the curve of the impedance of the GO-based sensor and transpiration rate under different irradiation intensities in a 3D coordinate. The curve of the GO-based sensor is nearly linear, and the impedance shows a negative linear correlation with irradiation intensities and transpiration rate. The calibration curve of transpiration sensing shows a good linear relationship in the trmmol range of 0–1 mmol m^−2^ s^−1^ (Figure S6), and the regression coefficient is 0.999. The result provides a possibility for detecting transpiration in growing crops using a GO-based sensor as the signal transducer. The plant growth rate is significantly affected by water status because water plays an important role in plant physiological processes. The GO-based wearable sensor technology would be useful to monitor the physiological water information of crops in real time and provide a convenient and effective tool for early monitoring and early warning of drought disasters. In the future, by studying the changes in water evaporation and net photosynthetic rate of crop leaves with light intensity, soil moisture, and other growth environments, as well as establishing the correlation, we can improve the understanding of the adaptability of crop physiological characteristics to growth environment, and therefore, reduce crop loss in the agricultural industry in the future. In addition, by using wireless sensor networks (WSNs) consisting of multiple plant sensors (Figure S7), smart and precision agriculture systems can be formed, providing an efficient solution to improve our capability of planting management.

## 4. Conclusions

In summary, a novel flexible GO-based noninvasive crop water sensor with high sensitivity, fast response rate and good biocompatibility was proposed. The sensor can harmlessly cohabitate with the plant and continuously track the water transportation in the plant, indicating the growth status, water consumption, and photosynthetic intensity. In situ real-time sensing of plant transpiration was realized, and the internal water transportation within maize plants could be monitored dynamically. Combining the GO-based sensor and photosynthesis measurement system, the synergistic effect of net photosynthetic rate and transpiration of plants under different light environments were revealed. All these demonstrate that the GO-based wearable sensor technology would be a convenient and effective tool for early monitoring and early warning of drought disasters in real time. By studying the changes in water evaporation and net photosynthetic rate of crop leaves with light intensity, other growth environments, as well as establishing the correlations, we can improve the understanding of the adaptability of crop physiological characteristics to the growth environment, and therefore, improve the crop productivity in the future. More generally, this study provides a new technical method to carry out quantitative monitoring of crop physiological information in the entire life cycle and to build a new intelligent planting system.

## Data Availability

Not applicable.

## Supplementary Materials

The following supporting information can be downloaded at: https://www.mdpi.com/article/10.3390/membranes12040358/s1, Figure S1: Elemental analysis of the material film by Energy Dispersive Spectrometer (EDS); Figure S2: The Raman spectra of rGO and GO. Figure S3: Hysteresis characteristic of the GO based sensor at 100 Hz; Figure S4: The fifteen-cycle response-recovery curve of GO based sensor. Figure S5: The long-term stability of the sensor. Figure S6: Calibration curve of maize plant leaf transpiration sensing; Figure S7: Schematic diagram of application scenario of the GO based flexible sensor for in situ real-time tracking of plant information.
