# Supplementary material for: Wearable Crop Sensor Based on Nano-Graphene Oxide for Noninvasive Real-Time Monitoring of Plant Water
Source: Membranes (Basel). 2022 Mar 24;12(4):358. doi: 10.3390/membranes12040358 (PMC9026295; doi:10.3390/membranes12040358)
Supplement: Supplementary file 1 [file membranes-12-00358-s001.zip › membranes-1568377-supplementary.pdf]

Supplementary Materials for

Wearable microscale crop leaf sensor based on nano-graphene oxide for noninvasive real time monitoring of physiological water status

Li Denghua[1,2,3], Li Ganqiong[1,2,3*], Li Jianzheng[1,2,3], Xu Shiwei[1,2,3*]

1 Agricultural Information Institute of Chinese Academy of Agricultural Sciences, Beijing 100081, China; lidenghua@caas.cn (L. D.); liganqiong@caas.cn (L.G.); lijianzheng@caas.cn (L. J.); xushiwei@caas.cn (X.S.)
2 Key Laboratory of Agricultural Information Service Technology, Ministry of Agriculture and Rural Affairs, Beijing 100081, China;
3 Research Center of Agricultural Monitoring and Early Warning Engineering Technology, Beijing 100081, China
* Correspondence: liganqiong@caas.cn (L.G.); xushiwei@caas.cn (X.S.)

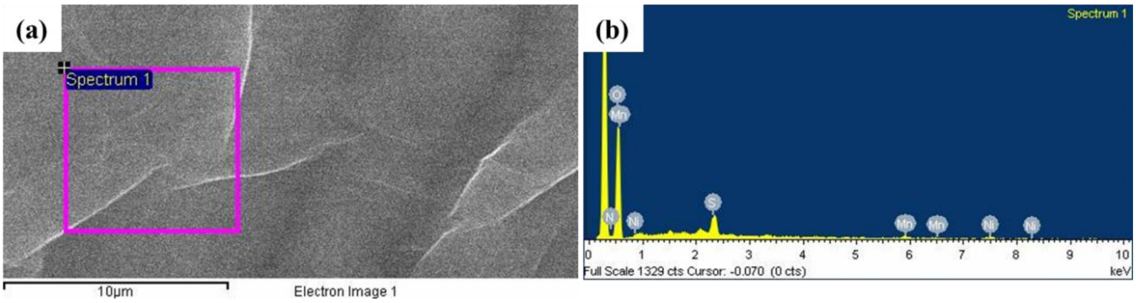

Fig. S1. Elemental analysis of the material film by Energy Dispersive Spectrometer (EDS).(a)SEM image of the graphene oxide film, pink inset shows the scan analysis area. (b) EDS image of the graphene oxide film. The content percentages of elements C and O were 60.1% and 36.4% respectively. The obtained GO sheet has a folded structure, and the high content of oxygen indicates that the functional groups are rich, which helps to make the GO sheets more hydrophilic and humidity sensitive.

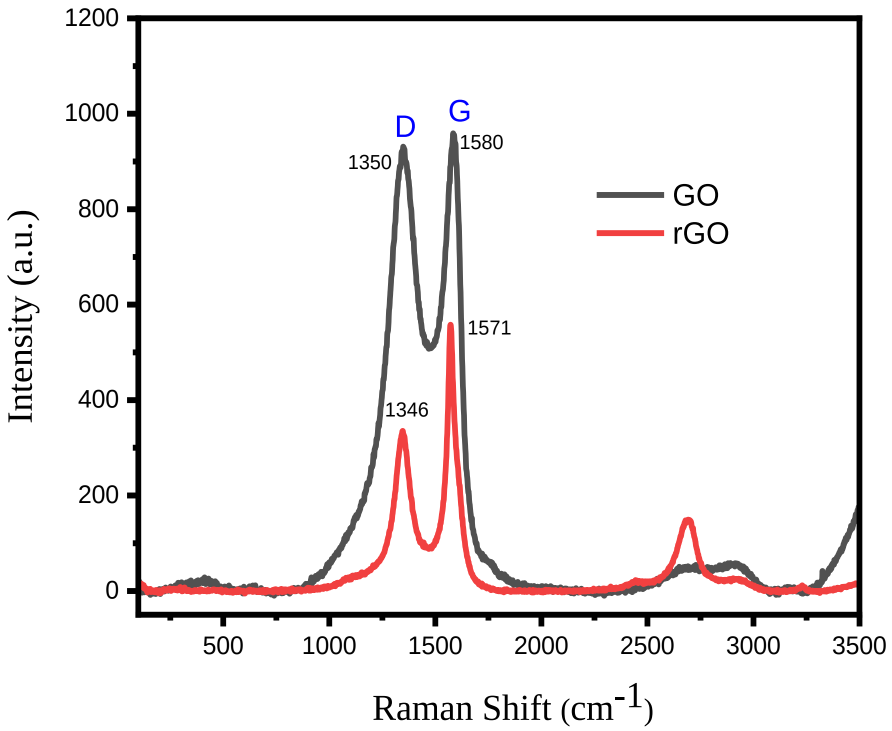

Fig. S2. The Raman spectra of rGO and GO. The I_D/I_G ratio of GO was 0.97, and the I_D/I_G ratio of rGO was 0.60. This implies that the defect ratio of GO is much higher than that of rGO.

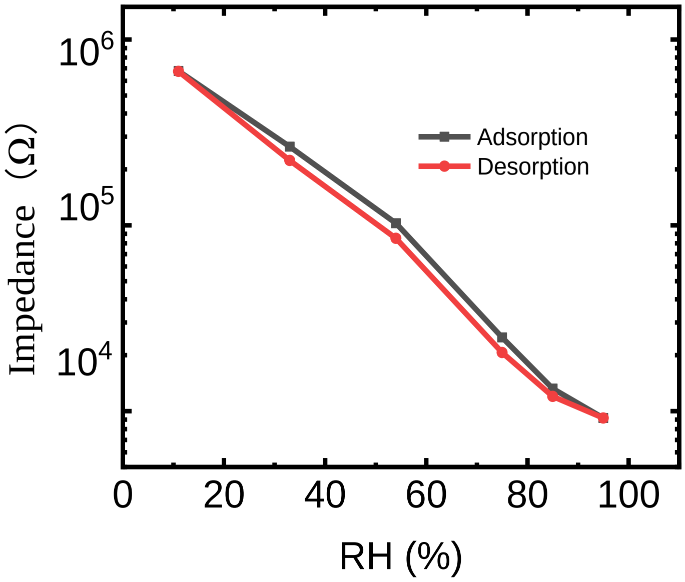

Fig. S3. Hysteresis characteristic of the GO based sensor at 100Hz.

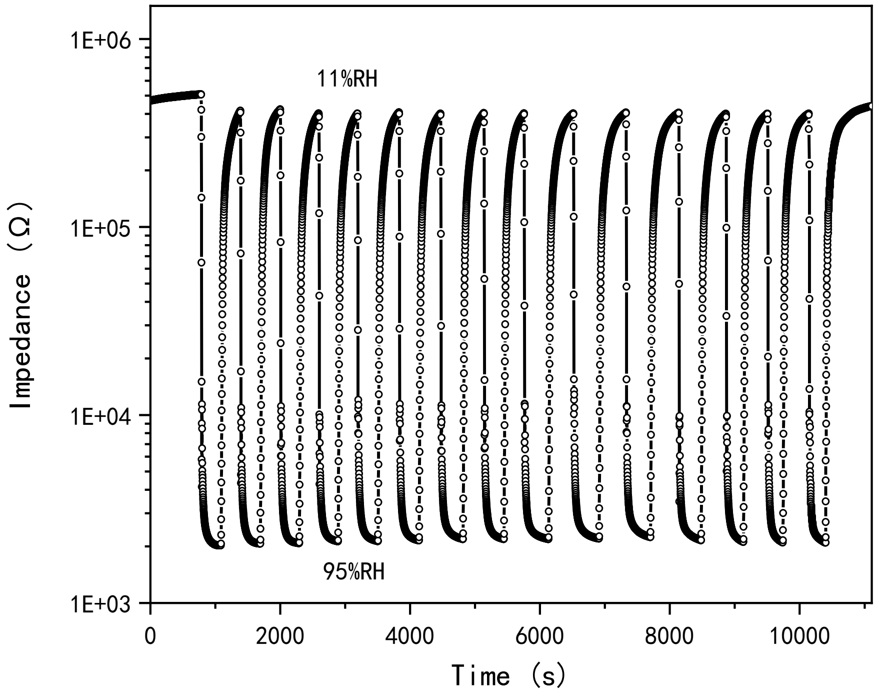

Fig. S4. The fifteen-cycle response-recovery curve of GO based sensor. Fifteen-cycle response-recovery experiments of GO based sensors were supplemented to verify the sensing repeatability

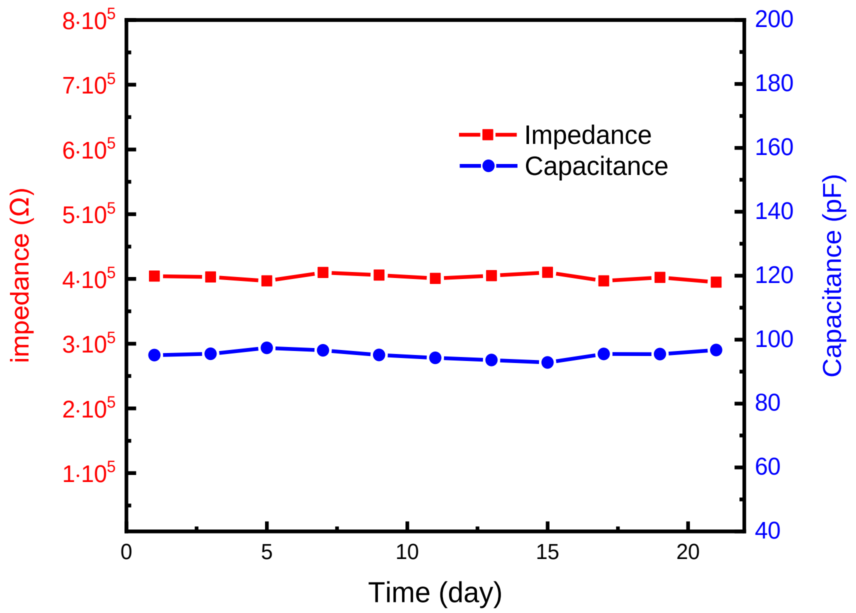

Fig. S5. The long-term stability of the sensor.

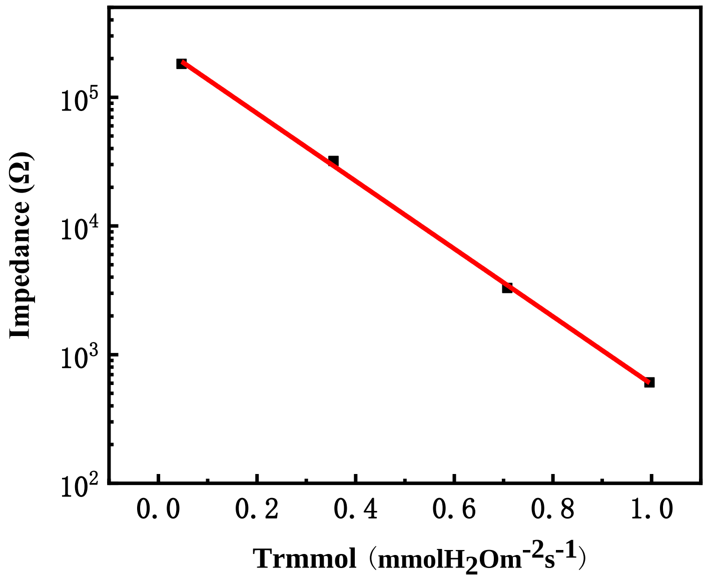

Fig. S6. Calibration curve of maize plant leaf transpiration sensing. The calibration curve of transpiration sensing shows a good linear relationship in the trmmol range of 0–1mmolm^{-2} s^{-1}. The functional relationship between sensor impedance response (Z) and transpiration rate (T) was fitted by the formula Z = a × T+ b, where a and b were constants. The response of the sensor showed a good linear relationship with transpiration rate (regression coefficient = 0.999). Due to the linear $Z - T$ relationship, this indicates that the GO based sensor as the signal transducer would hold great promise for detecting transpiration in growing crops.

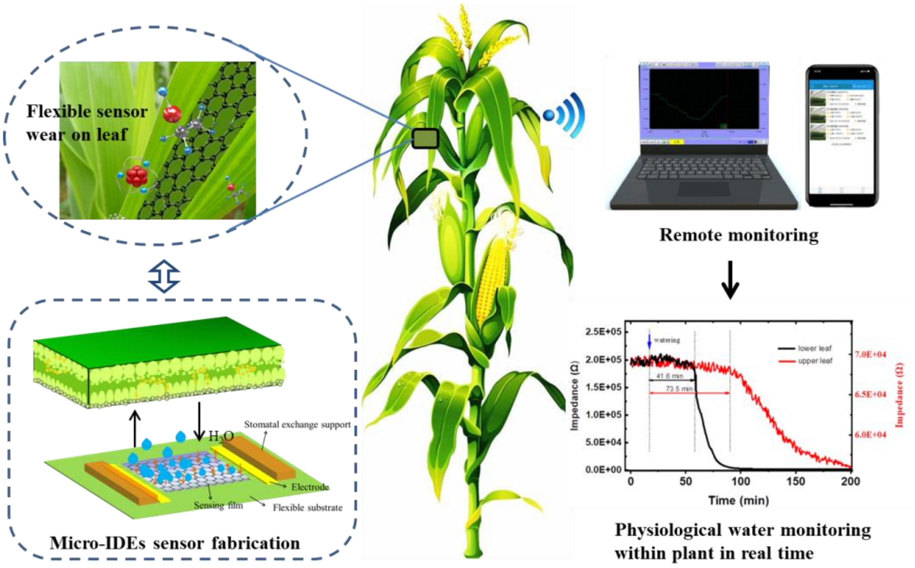

Fig. S7. Schematic diagram of application scenario of the GO based flexible sensor for in situ real-time tracking of plant information. The plant sensors can be attached on the surface of plants, which can form a smart agriculture system. Wireless communication is used to collect data from sensors and transmit the data information to a cellphone for wirelessly recording the real-time plant information.
